# Functional Analysis of the Cytoskeleton Protein MreB from *Chlamydophila pneumoniae*

**DOI:** 10.1371/journal.pone.0025129

**Published:** 2011-10-05

**Authors:** Ahmed Gaballah, Anna Kloeckner, Christian Otten, Hans-Georg Sahl, Beate Henrichfreise

**Affiliations:** University of Bonn, Institute for Medical Microbiology, Immunology and Parasitology, Pharmaceutical Microbiology Section, Bonn, Germany; Consejo Superior de Investigaciones Cientificas, Spain

## Abstract

In rod-shaped bacteria, the bacterial actin ortholog MreB is considered to organize the incorporation of cell wall precursors into the side-wall, whereas the tubulin homologue FtsZ is known to tether incorporation of cell wall building blocks at the developing septum. For intracellular bacteria, there is no need to compensate osmotic pressure by means of a cell wall, and peptidoglycan has not been reliably detected in *Chlamydiaceae*. Surprisingly, a nearly complete pathway for the biosynthesis of the cell wall building block lipid II has been found in the genomes of *Chlamydiaceae*. In a previous study, we discussed the hypothesis that conservation of lipid II biosynthesis in cell wall-lacking bacteria may reflect the intimate molecular linkage of cell wall biosynthesis and cell division and thus an essential role of the precursor in cell division. Here, we investigate why spherical-shaped chlamydiae harbor MreB which is almost exclusively found in elongated bacteria (i.e. rods, vibrios, spirilla) whereas they lack the otherwise essential division protein FtsZ. We demonstrate that chlamydial MreB polymerizes *in vitro* and that polymerization is not inhibited by the blocking agent A22. As observed for MreB from *Bacillus subtilis*, chlamydial MreB does not require ATP for polymerization but is capable of ATP hydrolysis in phosphate release assays. Co-pelleting and bacterial two-hybrid experiments indicate that MreB from *Chlamydophila (Chlamydia) pneumoniae* interacts with MurF, MraY and MurG, three key components in lipid II biosynthesis. In addition, MreB polymerization is improved in the presence of MurF. Our findings suggest that MreB is involved in tethering biosynthesis of lipid II and as such may be necessary for maintaining a functional divisome machinery in *Chlamydiaceae*.

## Introduction

The cytoskeletal protein MreB, a bacterial ortholog of actin, is widely distributed in rods, vibrios and spirilla and absent in most spherical bacteria [1], [2]. *Escherichia coli*, *Bacillus subtilis* and *Caulobacter crescentus mreB* knock-out mutants lose their typical shape and generate enlarged cells showing major morphological defects [3], [4], [5].

MreB belongs to the actin/Hsp70 superfamily, a functionally highly divergent group of proteins including heat shock proteins, sugar kinases and the plasmid stability protein ParM. They share limited amino acid identity but have a common fold that consists of two major symmetric domains folding around a nucleotide binding pocket [1]. Mg^2+^ or Ca^2+^ dependent ATP hydrolysis of these proteins is coupled to conformational changes which are known to regulate the activity of Hsp70 and the dynamic assembly of actin polymers [6].

MreB polymers are thought to tether incorporation of cell wall precursors into the side-wall during longitudinal growth [1] by recruiting and functionally organizing enzymes involved in cell wall precursor biosynthesis including soluble (MurB, MurC, MurE, MurF) and membrane (MraY and MurG) proteins [7]. The MreB organized enzyme machinery is connected to the PBP2 catalyzed precursor polymerization reactions on the outside through a membrane-spanning complex containing MreC, MreD and RodA [3], [7], [8]. The MreB protein self-assembles into filamentous polymeric structures *in vitro* and was considered to organize into helical filaments at the inner leaflet of the cytoplasmic membrane *in vivo* [1]. Based on total internal reflection fluorescence microscopy experiments, a recent study revealed that MreB assembles into discrete patches in *B. subtilis*, *E. coli* and *C. crescentus* [9]. A model for side-wall elongation in *B. subtilis* was proposed where MreB patches restricted the lateral diffusion of membrane-spanning wall elongation complexes to organize insertion of cell wall precursors along bands largely perpendicular to the long cell axis.

MreB polymers are dynamic structures that undergo cell cycle-related changes to reorganize into circumferential rings that flank the cytokinetic FtsZ ring. The cytoskeletal MreB rings are suggested to be involved in the division and segregation of the bacterial cytoskeleton and show interactions with MreC, MreD and RodA as well [10].

The tubulin ortholog FtsZ is almost ubiquitously distributed in bacteria, archaea and eukaryotic organelles [11] and is considered to be a central organizer of prokaryotic cell division. It assembles into an annular structure (Z-ring) at midcell and initiates cell division by attracting a set of proteins to form the cell divison machinery [11]. FtsZ is known to tether PBP3 (FtsI) catalyzed incorporation of cell wall building blocks at the developing septum [12].

Cell division and cell wall biosynthesis in prokaryotic cells are both driven by partially overlapping, tightly co-ordinated machineries. For intracellular bacteria such as *Chlamydiaceae*, there is no need to maintain osmotic stabilization by means of a cell wall, and peptidoglycan has not been reliably detected in *Chlamydiaceae* so far [13]. Nevertheless, antibiotics that target cell wall biosynthesis are also active against *Chlamydiaceae*, a paradox known as the chlamydial anomaly [13]. A nearly complete pathway for peptidoglycan biosynthesis has been found in the genomes of *Chlamydiaceae* [13], and activity of MurA, MurC/Ddl, CT390, DapF, MurE, MraY and MurG has been demonstrated [13], [14], [15], [16].

Recently, we discussed the hypothesis that maintaining lipid II biosynthesis in cell wall-less bacteria reflects an essential role of the lipid II biosynthesis pathway for prokaryotic cell division [16]. The aim of the present study is to gain understanding of the organization of lipid II biosynthesis in *Chlamydiaceae*. Surprisingly, these endobacteria lack the apparently essential division protein FtsZ, but harbor, despite their spherical shape, MreB [17].

Here, we demonstrate that chlamydial MreB polymerizes *in vitro* and interacts with key components in lipid II biosynthesis. Our findings suggest that MreB is involved in directing lipid II biosynthesis to the septum and as such may be necessary for maintaning a functional divisome machinery in *Chlamydiaceae*.

## Results

### MreB from *C. pneumoniae* polymerizes *in vitro*

**Figure 1:**
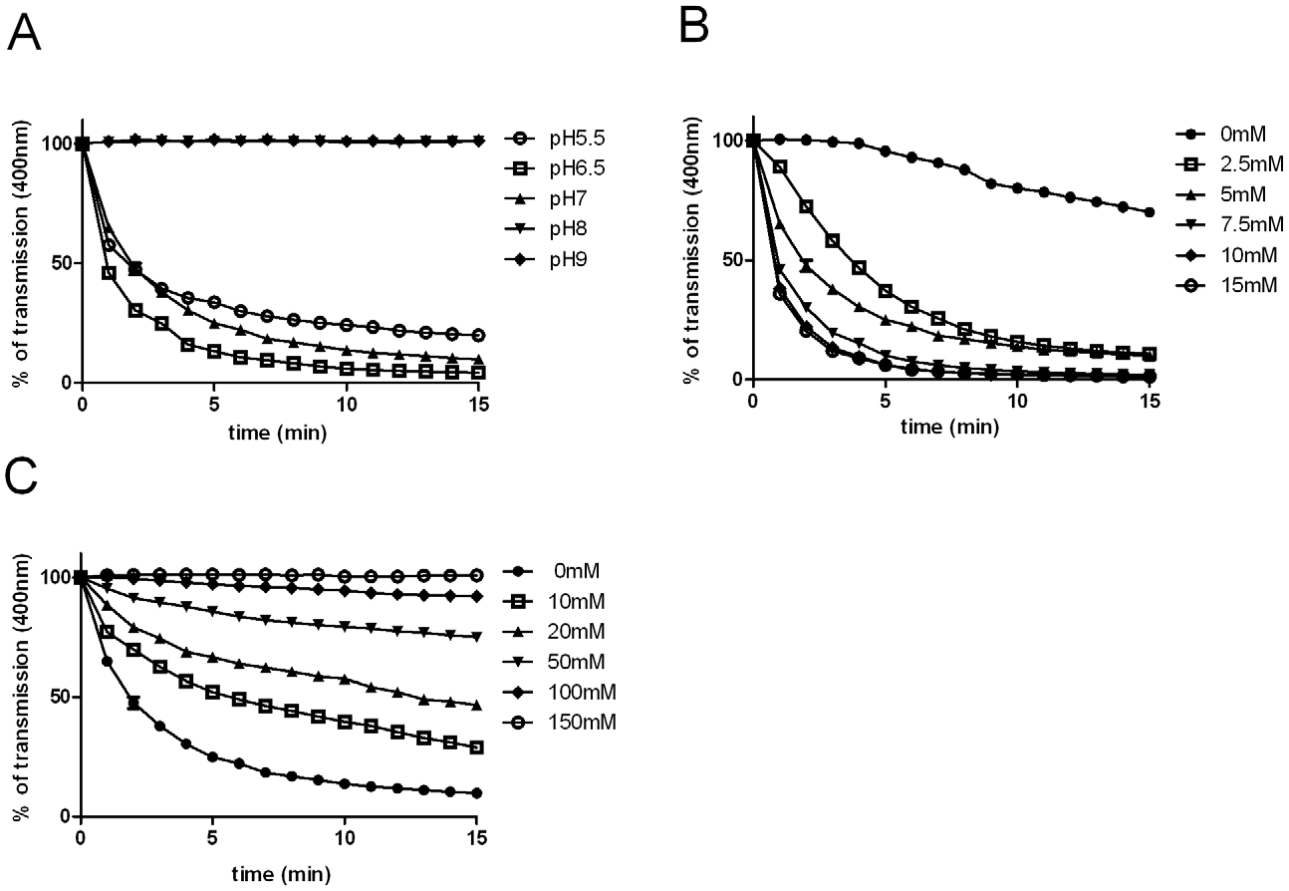
Polymerization of wild-type MreB from *C. pneumoniae*. *In vitro* light scattering assays were performed at varying pH (a) MgCl_2_ (b) and KCl (c) concentrations using 5 µM MreB.

**Figure 2:**
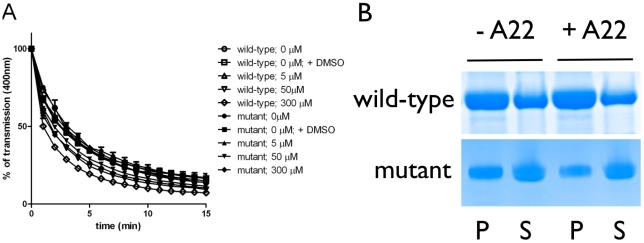
Influence of A22 on the polymerization of chlamydial MreB. The effect of A22 on MreB wild-type and C345A mutant polymerization was determined by light scattering (a) and sedimentation assays (b). For sedimentation assays, polymerized MreB proteins were centrifuged and equivalent volumes of supernatants and pellets were separated on a SDS-PAGE.

To examine the functionality of MreB from *C. pneumoniae*, we tested whether this protein can polymerize *in vitro*, as shown recently for MreB from *Thermotoga maritima* (MreB1) [18] and *Bacillus subtilis* [19]. We overproduced MreB from *C. pneumoniae* in *E. coli* and purified the recombinant protein. Chlamydial MreB polymerized *in vitro* in light scattering and sedimentation assays (Fig. 1 and Fig. 2b (left panel)). Polymerization was favored at low pH values and in the presence of Mg^2+^ ions but inhibited by K^+^ ions (Fig. 1 A–C). The chlamydial protein showed more similarity to MreB from *B. subtilis* than to MreB from *T. maritima* regarding its pH and K^+^ dependance. Polymerization of MreB from *C. pneumoniae* and *B. subtilis* was greatly impaired or completely inhibited at pH values of ≥8 and strongly inhibited at KCl concentrations of 20 mM. In contrast, MreB from *T. maritima* was shown to polymerize at pH as high as 9.5 and in presence of 20 mM KCl [18]. These properties probably reflect the adaptation of this extremophilic organism to geothermally heated marine sediments.

### Polymerization of MreB from *C. pneumoniae* is not inhibited by S-(3,4-Dichlorobenzyl)isothiourea (A22) *in vitro*

A22 is widespread applied to disrupt the cell cycle-related reorganization of the bacterial MreB cytoskeleton causing defects in cell morphology. The compound impairs the ATP dependent polymerization of *T. maritima* MreB *in vitro* by competitively binding into the nucleotide-binding pocket of the protein [20]. We investigated the effect of A22 on MreB polymerization using light scattering and sedimentation assays. Both experiments revealed that A22 did not impair the polymerization of chlamydial MreB *in vitro* (Fig. 2).

In a previous study [21], seven A22-resistant stable mutants from *Caulobacter crescentus* were generated under A22 selection pressure. All mutants showed amino acid exchanges in MreB which could be mapped to the nucleotide-binding pocket of the protein and affected residues that were conserved in MreB from *C. crescentus*, *T. maritima* and *E. coli*. One of these altered residues in mutant *C. crescentus* MreB is intrinsically exchanged in wild-type MreB from *C. pneumoniae* (Ala345 located in helix 12 is replaced by cysteine, numbering of *C. pneumoniae*). To test whether this natural alteration in chlamydial wild-type MreB is responsible for insusceptibility to A22, we replaced Cys345 with alanine and investigated the effect of A22 on the mutant protein. Interestingly, the C345A mutant showed the same phenotype like the wild-type protein in that its polymerization was not affected by A22 (Fig. 2b).

### *In vitro* polymerization of C345A mutant and wild-type MreB proteins is independent of the presence of ATP

**Figure 3:**
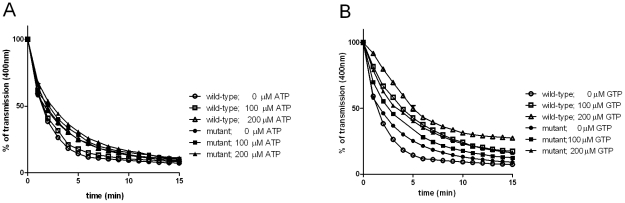
Influence of nucleotides on MreB polymerization. Polymerization of chlamydial wildtype and C345A mutant MreB was investigated in the presence and absence of ATP (a) and GTP (b) using light scattering assays under standard conditions.

To further analyze the insusceptibiliy of chlamydial MreB to A22, we tested whether chlamydial MreB might be capable of polymerizing in the absence of nucleotides (Fig. 3). Remarkably, both the C345A mutant and wild-type MreB proteins polymerized in the same time course and to the same extent in the absence or presence of ATP. To exclude that MreB-bound, co-purified nucleotides might have driven polymerization independent from the addition of ATP, we treated the purified MreB wild-type and C345A mutant protein solutions with an anion exchange resin prior to use, as described previously for nucleotide exchange procedures for MreB [18], [19]. The so-treated MreB species still polymerized without the addition of ATP (data not shown).

Therefore, it appears reasonable that a competitive inhibitor of nucleotide binding such as A22 cannot block the polymerization of chlamydial MreB proteins. In the presence of GTP, the kinetics and extent of wild-type MreB polymerization was impaired (Fig. 3b). Binding of GTP to the nucleotide binding pocket of chlamydial MreB might introduce conformational changes that impact on the polymerization process. Our findings on ATP independent polymerization are consistent with previous *in vitro* results on MreB from *Bacillus subtilis* which showed that the purified actin ortholog polymerized in a nucleotide independent fashion [19].

### C345A mutant and wild-type MreB proteins from *C. pneumoniae* hydrolyze ATP *in vitro*

**Figure 4:**
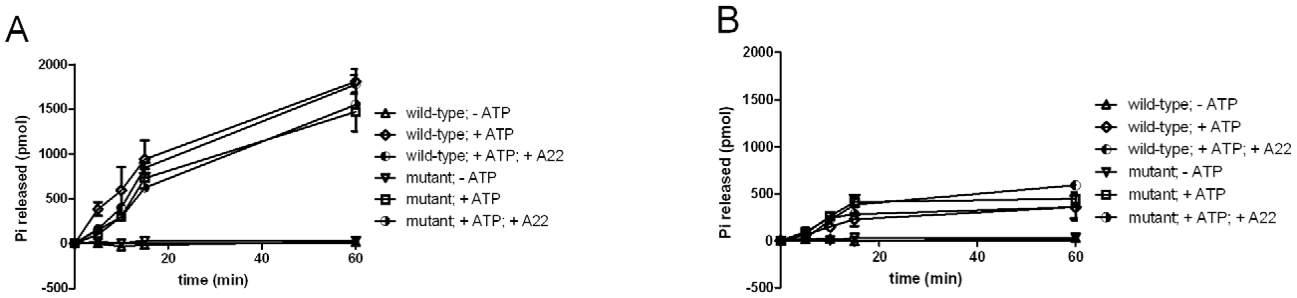
ATPase activity of wild-type and C345A mutant MreB under monomeric (A) and polymerizing (B) conditions. To quantify ATP hydrolysis phosphate release was measured using a molybdate∶malachite green assay. Wild-type and C345A mutant MreB (5 µM) were incubated with ATP (200 µM) under standard polymerizing and non-polymerizing conditions.

MreB from *B. subtilis* does not require nucleotides for polymerization but can hydrolyze ATP [19]. To test whether chlamydial MreB is a functional ATPase, we performed phosphate release assays (Fig. 4). The C345A mutant and wild-type MreB proteins hydrolyzed ATP without marked differences. Using buffer conditions that do not allow polymerization, monomeric MreB hydrolysed ATP continuously during the whole time of the experiment (1 h). In contrast, under standard polymerization conditions ATPase activity was stalled once polymerization of MreB was completed (after 15 min), and phosphate release reached only approximately one third of the level measured for monomeric MreB. These results indicate that ATPase activity is associated with the monomeric stage of MreB.

A22 had no inhibitory effect on ATP hydrolysis in all experiments. This indicates that the compound cannot bind to the nucleotide binding pocket of both the wild-type and the C345A mutant MreB.

### Chlamydial MreB interacts with MurF, MraY and MurG

We examined the interactions of MreB with MurF, MraY and MurG, three key enzymes involved in cell wall biosynthesis. The ligase MurF attaches the dipeptide D-Ala-D-Ala to the UDP-N-acetylmuramic acid-tripeptide precursor. MraY catalyses the synthesis of the first membrane bound intermediate by transfering N-acetylmuramic acid-pentapeptide to the lipid carrier undecaprenyl phosphate yielding lipid I. Subsequently, MurG adds N-acetylglucosamine yielding the final peptidoglycan precursur lipid II.

**Figure 5:**
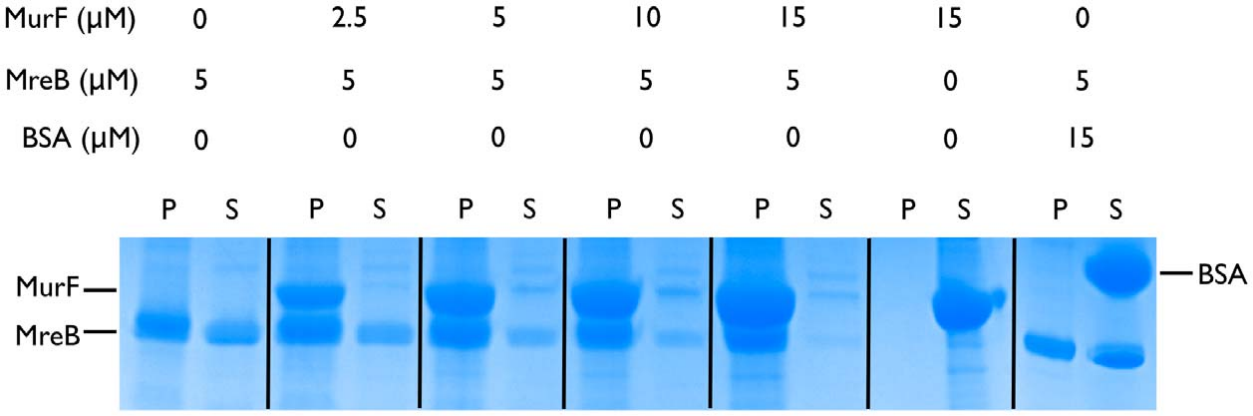
*In vitro* interaction of MreB and MuF from *C. pneumoniae* in co-pelleting assays. For sedimentation assays, MreB was polymerized in the absence and in the presence of increasing amounts of MurF. After centrifugation, equivalent volumes of supernatants (S) and pellets (P) were separated on a SDS-PAGE.

We detected interaction of MreB with MurF *in vitro* using a co-pelleting assay that monitors for MreB polymerization in the presence of MurF. MurF co-pelleted with MreB polymers and did not pellet on its own (Fig. 5). In a control containing BSA and MreB, BSA remained in the supernatant and did not interact with MreB polymers in a non-specific manner. Keeping the concentration of MreB (5 µM) constant and increasing the concentration of MurF (2.5 µM, 5 µM, 10 µM, 15 µM), MreB polymerized to an increasing extent and spun down in the pellet. At a molar ratio of 1∶3 (MreB∶MurF), roughly all MreB polymerized and was recovered in the pellet. These results indicate that polymerization of MreB is favored in the presence of MurF.

**Figure 6:**
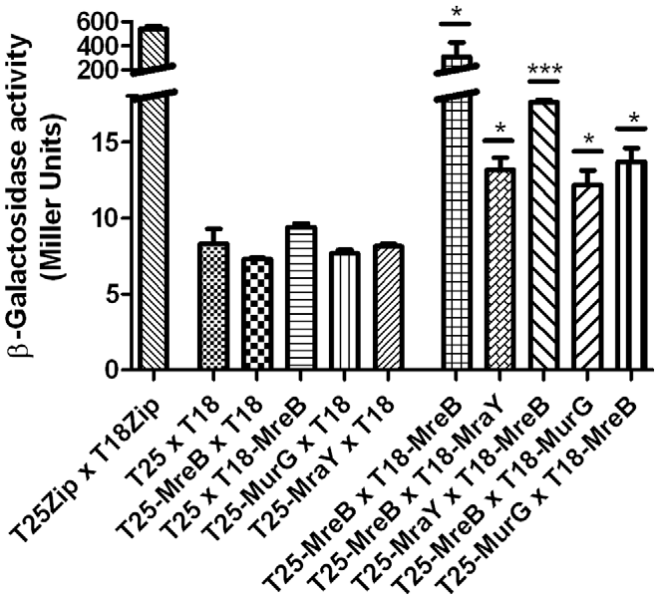
Bacterial two hybrid studies on *in vivo* interactions between chlamydial MreB, MraY and MurG. Protein interactions were quantitatively determined by measuring β-galactosidase activities of three individually selected replicate cotransformants. The β-galactosidase activities are expressed in Miller Units (change in A420/min/ml/OD_600_) as mean values (± standard deviation). Unpaired t-test revealed statistical significance in comparision to empty vector negative control (T25×T18), *: two-tailed p-value≤0.05, *** two-tailed p-value≤0.001).

A bacterial adenylate cyclase two-hybrid system (BACTH system, Euromedex, France) was used for analysing interactions between MreB, MraY and MurG *in vivo*. Bacterial two-hybrid analysis confirmed our results on *in vitro* polymerization of MreB in that self-interaction was detected (Fig. 6). Additionally, we found MreB interacting with both the integral membrane protein MraY and the membrane associated cytosolic MurG. As expected from the localisation of the N-terminal adenylate cyclase fragments, these interactions showed relatively low β-galactosidase activities. In fusion with MraY, the adenylate cyclase fragment was located in the periplasm (predicted from topology studies [22], and using topology prediction program TMHMM [23]). Fused with MurG, the respective fragment was located closed to the membrane association site of the protein (predicted from the crystal structure [24]).

## Discussion

Like many other bacteria occupying a defined and narrow ecological niche (e.g., *Mycoplasma pneumoniae*, [25], *Rickettsia conorii* [26]), all members of *Chlamydiaceae* have a remarkable reduced genome size [17]. In the course of adaptation to the isotonic host cell enviroment they apparently could afford to lose a functional cell wall. They also lost the cell division driving FtsZ but retained the cytoskeletal protein MreB and a lipid II biosynthesis pathway. Cell division and cell wall biosynthesis in prokaryotes are driven by partially overlapping multiprotein machineries whose activity needs to be tightly co-ordinated to maintain cell integrity. Recently, we proposed that conservation of lipid II biosynthesis in cell wall-lacking endobacteria may reflect the intimate molecular linkage of cell wall biosynthesis and cell division and thus a vital role of the bactoprenol bound precursor in cell division [16]. In this study, we investigated whether MreB plays a role in organizing lipid II biosynthesis in *Chlamydiaceae*. We showed that chlamydial MreB interacts with MurF, MraY and MurG, three key components of lipid II biosynthesis. Additionally, MreB was functional and polymerized *in vitro*. Polymerization of chlamydial MreB was favored in the presence of MurF but, as shown recently for *B. subtilis* MreB [9], strongly inhibited at K^+^ concentrations as low as 20 mM. *In vivo* studies proved that MreB from *B. subtilis* is able to polymerize under physiological conditions (e.g. [9], [27]) with K^+^ concentrations of about 300 mM [28]. Interplay of enhancing mechanisms (protein interaction) and inhibitory mechanisms (salt susceptibility) may contribute to modulate and control the polymerization of MreB *in vivo*.

Chlamydial MreB and MurG interact with each other (this study). MurG is functionally conserved in *C. pneumoniae* and transfers N-acetylglucosamine onto lipid I yielding lipid II *in vitro* [16]. Moreover, these two proteins have been shown to localize at the division site in *E. coli* and *C. crescentus* [10], [29], [30]. Based on our recent hypothesis that lipid II biosynthesis may have to be maintained for functional cell division [16], MreB could be involved in maintaining co-ordinated cell division via tethering the biosynthesis of lipid II to the chlamydial septum.

Our hypothesis regarding chlamydial MreB is supported by a recent study on L-forms of *Bacillus subtilis* [31]. L-form strains are wall-deficient spherical derivatives of common bacteria which divide indefinitely. The stable L-forms generated in the study by Leaver et al. did not require FtsZ for cell-division [31]. These authors postulated a FtsZ independent extrusion-resolution mechanism in which a cytoskeletal system (such as builded up by *B. subtilis* actin orthologs MreB, Mbl or MreBH) or an active chromosome segregation system might provide active force generation. L-form mutants which lacked a D-Alanine racemase that converts L-Alanine into D-Alanine could grow in the absence of D-Alanine, and electron microscopy studies revealed no hints of residual cell wall outside the boundary of the cytoplasm or at the division site. Because chlamydiae also lack a cell wall and might not be able to synthesize D-Alanine in absence of a D-Alanine racemase, these findings are in good agreement with our previous hypothesis that the monomeric precursor lipid II – possibly with an uncommon structure of the peptide-side chain lacking D-Alanine – is required for cell division in *Chlamydiaceae* [16].

It appears that an entire cycle of lipid II biosynthesis and processing, including translocation to the outside and recycling of the bactoprenol carrier, needs to be maintained for co-ordinated function of the divisome machinery. The fate of lipid II in chlamydiae is unclear. These endobacteria encode the flippase FtsW, an integral membrane protein which translocates the precursor across the membrane [17], [32]. Moreover, they harbor two genes encoding monofunctional transpeptidases (PBP2 and PBP3 (FtsI)) [17], which cross-link glycan chains via peptide bridges in free-living bacteria. They also encode a D-alanyl-D-alanine carboxypeptidase (PBP6a) and a N-acetylmuramyl-L-alanine amidase (AmiA) [17] which cleaves peptide side chains from glycan chains during cell division in extracellular bacteria. The intracellular *Chlamydiaceae* lack transglycosylases and endopeptidases that are needed to link the sugar units of lipid II to form glycan chains or to cleave peptide bridges between cross-linked glycan strains, respectively [17]. In addition, they do not posess pyrophosphorylases which catalyze in *E. coli* bactoprenol-P recycling by dephosphorylation of bactoprenol-PP [33]. Moreover, chlamydial genomes do not encode MreD or MreC, two proteins which are in elongated bacteria encoded in one operon together with MreB [17]. During longitudinal growth, MreC and MreD play a crucial role in connecting the MreB organized precursor biosynthesis machinery inside the cell with the PBP2 catalyzed incorporation of cell wall precursors on the outside [7]. For the spherical bacteria, shape-maintaining functions and spacial organizing of PBP2 reactions are dispensible. Therefore, chlamydiae may have not retained MreC and MreD.

**Figure 7:**
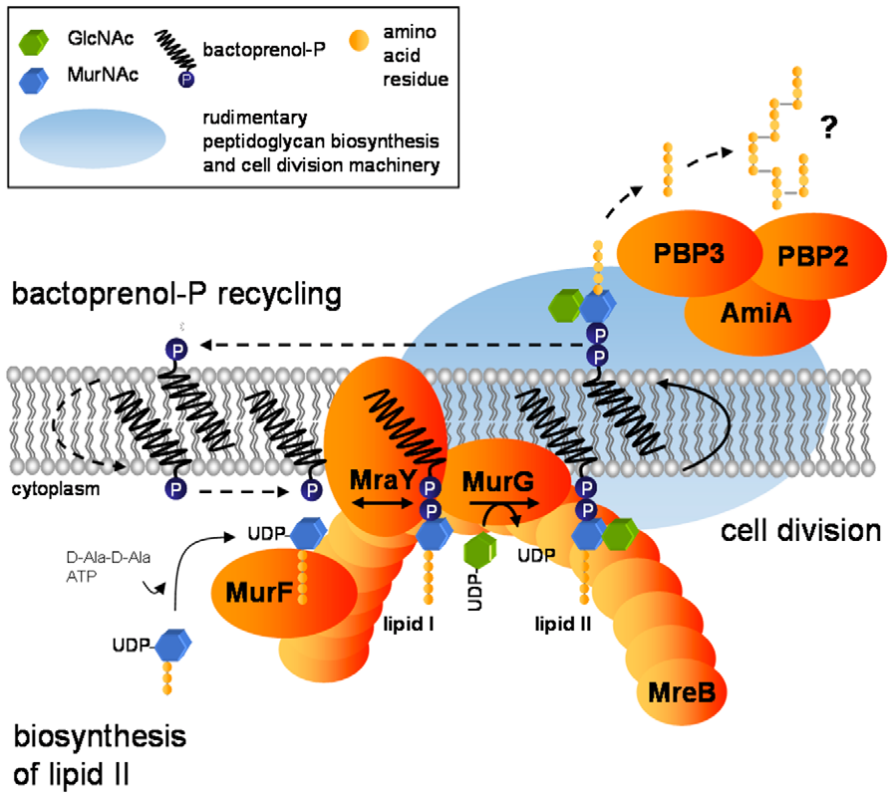
Proposed model for co-ordination of lipid II biosynthesis and cell division in chlamydiae. A complete cycle of lipid II biosynthesis and processing, including translocation to the outside and recycling of the bactoprenol carrier, needs to be maintained for co-ordinated function of the cell division machinery (which may consist of FtsW, FtsI, FtsH, FtsK and FtsY). The actin ortholog MreB functionally organizes MurF, MraY and MurG and directs lipid II biosynthesis to the septum. The synthesized precursor is translocated to the outside where the release of the bactoprenol carrier is catalyzed through the activity of FtsI and PBP2 (as well as probably AmiA). Question marks and dashed arrows indicate steps of the proposed pathway that remain to be elucidated. (GlcNAc: N-acetylglucosamine; MurNAc: N-acetylmuramic acid).

We propose the following model for a linkage between lipid II biosynthesis and cell division in chlamydiae (Fig. 7).

MreB interacts with MurF, MraY and MurG and serves as a scaffold directing lipid II biosynthesis to the septum. The synthesized precursor is processed by a rudimentary cell wall biosynthesis cell division machinery. First, translocation of lipid II across the membrane is catalyzed by flippase FtsW. Subsequently, PBP2 and FtsI (as well as probably AmiA) catalyze the release of the bactoprenol carrier. In the process, a rudimentary (glycan-less [34]) by-product in which the peptide sidechains are cross-linked by peptide bonds might result. Recycling of bactoprenol-P remains unclear in the absense of pyrophoshorylases that have been described so far to dephoshorylate bactoprenol-PP.

Of note, a recent study showed that in reticulate bodies, the replicative form of chlamydiae, cell division is arrested by the second binary fission in the presence of penicillin [35]. Binding of penicillin to chlamydial PBPs may interrupt processing of lipid II and cell division might be arrested due to depletion of the bactoprenol carrier.

Functional analysis of the chlamydial PBPs and AmiA, as well as determination of the exact structure of lipid II will be necessary to further understand the processing of the precursor in chlamydiae.

MreB from *C. pneumoniae*, *B. subtilis*, and *T. maritima* show more than 54% of amino acid identities. Despite these high sequence similarities, these proteins differ regarding requirements on nucleotides for polymerization their susceptibility towards A22. All actin orthologs are functional ATPases. However, in contrast to MreB from *T. maritima*, the proteins from *B. subtilis* and *C. pneumoniae* polymerize in the absence of ATP (this study, [18], [19]).

A22 is frequently used for *in vivo* studies on the bacterial MreB cytoskeleton. This compound could be identified *in vitro* as a competitive inhibitor of ATP binding to MreB from *T. maritima* [20]. The minimal inhibitory concentration (MIC) of A22 towards *B. subtilis* is extremely high (100 mg/L) and almost as high as towards *S. aureus* (>100 mg/L), a species lacking MreB [36]. In accordance with our findings on ATP independent polymerization, A22 did not inhibit polymerization of chlamydial wild-type and C345A mutant MreB *in vitro*. Additionally, A22 did not inhibit ATP hydrolysis indicating that the compound cannot bind to the nucleotide binding pocket of MreB from *C. pneumoniae*. *In vitro* experiments on the polymerization and the ATPase activity of *B. subtilis* MreB in presence of A22 may be helpful to elucidate, whether the *B. subtilis* protein is not a target of A22, as shown in this study for MreB from *C. pneumoniae*.

A22 affects bacterial growth not only by acting on MreB but also on (an)other essential unknown target(s). This has been shown in several recent reports on (i) *E. coli* mutants that lack MreB, (ii) an A22 resistant *Anabaena* mutant strain retaining a wild-type *mreB gene*, and (iii) rod-shaped bacteria containing (*C. crescentus*) or not containing MreB (*Sinorhizobium meliloti*, *Agrobacterium tumefaciens*) [37], [38]. Further studies dealing with the *in vivo* effect of A22 on chlamydiae, bacteria with small genomes and A22-insusceptible MreB, might be useful for the identification of these unknown targets.

Orchestration of lipid II biosynthesis and processing as well as cell division may be achieved by formation of multi-enzyme complexes. Additional studies on the interaction of MreB with proteins involved in these fundamental processes will be helpful for a better understanding into the role of MreB in functionally organizing lipid II biosynthesis at the septum and as such in maintaining a functional division machinery. Deeper insight in co-ordination of lipid II biosynthesis and cell division will contribute to elucidate the inscrutable chlamydial anomaly at a molecular level. In addition, it will provide a basis for the design of new antibacterial drugs to combat diseases caused by these major intracellular pathogens.

## Materials and Methods

### Cloning of chlamydial *mreB*

The *mreB* gene from *C. pneumoniae* GiD [39] was amplified by PCR using the primers mreB-1 (5′-gatgaattcagtccacatcgcaatctg-3′) and mreB-2 (5′-ccactcgagtaccaaattccctttacg-3′) (restriction sites are underlined). The PCR product was digested by EcoR1 and XhoI and ligated into the EcoR1/XhoI site of the vector pET-21b (Novagen, VWR International, Darmstadt, Germany) such that MreB carried a C-terminal His_6_ tag. The correctness of the *mreB* sequence was confirmed by sequencing.

### Site-directed mutagenesis

Cys345 in chlamydial MreB was changed to alanine using the QuikChange Lightning Site-Directed Mutagenesis Kit (Agilent Technologies, Waldbronn, Germany). The primers mreB-C345A-sense (5′-catcctttgctggcagttgctttaggaaccgggaaagc-3′) and mreB-C345A-antisense (5′-gctttcccggttcctaaagcaactgccagcaaaggatg-3′) (C345A resulting base changes are underlined) were used according to the manufacturers' instructions. The correct base changes were confirmed by sequencing.

### MreB overproduction and purification

For the overproduction of wild-type and C345A mutant MreB proteins, the appropriate vector was transformed into *E. coli* BL21(DE3) (New England Biolabs, Frankfurt, Germany). Cultures were grown at 30°C in LB broth supplemented with 100 mg/L ampicillin. At an optical density (OD_600_) of 0.5–0.6, induction was achieved by the addition of IPTG to a final concentration of 0.5 mM and incubation for 4 h at 30°C. Cells were harvested by centrifugation (7,000× g for 10 min at 4°C) and stored at −20°C. The cell pellet of one liter culture was suspended in 6 ml lysis buffer (50 mM Tris-HCl, 300 mM NaCl, pH 8). Lysozyme was added to a final concentration of 0.5 mg/ml and cell suspension was incubated at room temperature for 30 min followed by sonication and centrifugation (15,000× g for 30 min at 4°C). For purification of the His-tagged protein from the supernatant, the cleared lysate was incubated with 1 ml of washed and equilibrated Ni^2+^-nitrilotriacetic acid (Ni-NTA) resin (Qiagen, Hilden, Germany) for 1 hour at 4°C. The Ni-NTA resin was then packed into gravity-flow columns, washed two times with 5 ml lysis buffer containing 10 mM and 20 mM imidazole, respectively. His-tagged protein was eluted using 3 ml of elution buffer (50 mM Tris-HCl, 300 mM NaCl, 250 mM imidazole, pH 8) and collected in 0.5 ml fractions. The purified proteins were analyzed by SDS-PAGE stained with Coomassie blue. The elution fractions containing the proteins were dialysed against 5 mM Tris-HCl buffer, pH 8 for at least 30 hours. The wild-type and C345A mutant MreB proteins were quantified using Bradford.

### MreB light scattering assays

Polymerization of MreB was investigated using light scattering assays described previously by Bean and Aman [18] with slight modifications. The standard polymerization reaction contained 5 µM MreB, 200 µM ATP, 5 mM MgCl_2_, 1 mM EGTA, and 10 mM imidazole buffer, pH 7 in a final volume of 800 µl. The non-protein components were mixed. Separately on ice, MreB was mixed with 1/9^th^ volume of 10× cation exchange buffer (1 mM MgCl_2_, 10 mM EGTA) and incubated for 1 min. Polymerization reaction was initiated by combining the two solutions and performed at 20°C for 15 min. Polymerization was detected by quantifying percentage of transmission at 400 nm in a spectrophotometer. All experiments were performed in triplicate. For A22 inhibition studies, A22 was dissolved in DMSO and added to the non-protein part of the polymerization reaction in a final concentration of 5, 50 and 300 µM.

### MreB sedimentation assays

Experiments followed the protocol of Bean and Amann [18]. Briefly, MreB (5 µM) was polymerized under standard conditions in presence and absence of 300 µM A22 for 1 hour at 20°C. Polymerized MreB was centrifuged and equivalent volumes of pellet and supernatant were analyzed by SDS-PAGE and Coomassie Blue.

### MreB and MurF co-pelleting assays

*In vitro* interaction of MreB with MurF was investigated following a previously described copelleting protocol [40].

The sedimentation assays described above were performed with MreB (5 µM) polymerized in presence and absence of 5 µM chlamydial MurF. Controlling precipitation of MurF or unspecific protein interactions, MurF (15 µM) was tested in the absence of MreB, and MreB was tested in the presence of BSA (15 µM), respectively. After centrifugation, supernatants were removed for analysis and pellets were washed with polymerization buffer. Equivalent volumes of pellet and supernatant were analyzed by SDS-PAGE and Coomassie Blue.

### Phosphate release assays

To analyse ATPase activity of MreB we investigated phosphate release using a molybdate∶malachite green assay [41]. Wild-type and C345A mutant MreB were incubated with ATP using standard polymerizing and non-polymerizing (in the absence of MgCl_2_, pH 8) conditions. Control reactions without ATP and without MreB were done as well. After different time intervals, 100 µl aliquots were removed and mixed directly with 5 µl ice cold 70% perchloric acid (Merck, Darmstadt, Germany) to stop the enzymatic reaction. The so treated reaction was incubated on ice for 10 minutes and centrifuged for 5 minutes at 49,500× g, 4°C. The supernatant was mixed with equal volume of Molybdate Dye Solution (Promega, Mannheim, Germany) and incubated in microtiter plate for 30 minutes at room temperature. Absorbance was measured at 600 nm in a spectrophotometer. All experiments were carried out in triplicate.

### Bacterial two-hybrid interaction studies

A bacterial adenylate cyclase two-hybrid system (BACTH system, Euromedex, France) was used for *in vivo* interaction studies between MreB, MraY and MurG. Briefly, the T25 and T18 fragments of the catalytic domain of *Bordetella pertussis* adenylate cyclase were fused to full-lengh copies of each protein. Each interaction was checked twice with both fusion protein combinations. For each combination, protein interactions were quantitatively determined by measuring beta-galactosidase activities of three individually selected replicate co-transformants. Additionally, we monitored the background activity due to interactions of the chlamydial proteins with the T25 and T18 fragments.
